# Efficacy of Combined Cervical Pessary and Progesterone in Women at High-Risk of Preterm Birth †

**DOI:** 10.3390/diagnostics16030402

**Published:** 2026-01-27

**Authors:** Marcelo Santucci França, Gabriela Ubeda Santucci França, Alan Roberto Hatanaka, Evelyn Traina, Tatiana Emy Kawanami Hamamoto, Danilo Brito Silva, Edward Araujo Júnior, Rosiane Mattar, Antonio Braga, Rodolfo de Carvalho Pacagnella

**Affiliations:** 1 Department of Obstetrics, Paulista School of Medicine-Federal University of São Paulo (EPM-UNIFESP), São Paulo 04023-062, SP, Brazil; m.franca22@unifesp.br (M.S.F.); gabi.ubeda1410@gmail.com (G.U.S.F.); alan.hatanaka@unifesp.br (A.R.H.); evelyntraina@gmail.com (E.T.); tatikawanami@gmail.com (T.E.K.H.); danilo.brito@unifesp.br (D.B.S.); araujojred@terra.com.br (E.A.J.); rosiane.mattar@unifesp.br (R.M.); 2 Department of Gynecology and Obstetrics, School of Medicine, Federal University of Rio de Janeiro (UFRJ), Rio de Janeiro 22240-003, RJ, Brazil; 3 Department of General and Specialized Surgery, School of Medicine and Surgery, Federal University of the State of Rio de Janeiro (UNIRIO), Rio de Janeiro 20271-062, RJ, Brazil; 4 Postgraduate Program in Applied Health Sciences, University of Vassouras, Vassouras 27700-000, RJ, Brazil; 5 Department of Obstetrics and Gynecology, State University of Campinas (UNICAMP), Campinas 13083-859, SP, Brazil; rodolfop@unicamp.br

**Keywords:** preterm birth, cervical pessary, vaginal progesterone, previous preterm birth, short cervical length, predictive factors

## Abstract

**Objective:** This study assessed the efficacy of the cervical pessary combined with progesterone to prevent preterm birth in pregnant women with short cervix and previous preterm birth. **Methods:** This post hoc analysis of the randomized, multicenter P5 trial examined the efficacy of the cervical pessary associated with vaginal progesterone versus progesterone alone for preventing recurrent preterm birth in 155 pregnant women with cervical length ≤30 mm and prior spontaneous preterm birth (sPPTB) (main subgroup), and in 85 women with cervical length ≤25 mm and sPPTB (higher-risk population). The primary outcome was spontaneous preterm birth (sPTB) before 34 weeks; secondary outcomes included sPTB rates before 37, 32, and 28 weeks, analyzed using Odds Ratio (OR) and Kaplan–Meier curves. A secondary objective was to identify predictive factors for sPTB recurrence in the cohort with prior preterm birth (n = 479), irrespective of treatment allocation. **Results:** Demographic profiles were balanced between groups. The addition of a cervical pessary to progesterone did not result in a significant reduction in sPTB before 34 weeks: to cervix ≤30 mm, OR 1.169 (95% CI 0.524–2.609; *p* = 0.703) and 1.167 (95% CI 0.466–2.921; *p* = 0.742) for ≤25 mm; similar null findings were observed across all gestational age thresholds. Kaplan–Meier survival curves demonstrated no significant differences between groups (*p* > 0.05). Secondary analysis (n = 479) identified principal predictors of sPTB recurrence, regardless of the cervical length: higher education (OR 2.37; 95% CI 0.99–5.63; *p* = 0.024), previous cervical conization (OR 4.78; 95% CI 1.08–21.19; *p* = 0.039) previous low birth weight < 2.5 kg (OR 2.43; 95% CI 1.22–4.85; *p* = 0.051), prior miscarriages (OR 1.36; 95% CI 1.10–1.69; *p* = 0.005), current twin pregnancy (OR 14.86; 95% CI 4.35–50.68; *p* < 0.001) and cervical funneling (OR 3.60; 95% CI 1.79–7.24; *p* < 0.001). Predictive models achieved an AUC of 0.719, with 87.0% sensitivity and 58.8% specificity. **Conclusions:** These findings do not support the routine use of cervical pessary combined with progesterone in women with dual risk factors. In this Brazilian population, specific clinical and obstetric characteristics—including higher education, cervical funneling, prior low birth weight delivery, previous conization, current twin gestation, and prior miscarriage—could identify women at increased risk for recurrent preterm birth.

## 1. Introduction

Preterm birth, defined as birth before 37 weeks of gestation weeks, is a leading cause of global neonatal morbidity and mortality, affecting more than 15 million births annually and accounting for 35% of neonatal deaths. Survivors face long-term sequelae including cerebral palsy and chronic respiratory complications [1,2]. Brazil has among the highest preterm birth rates globally (11–12%), imposing substantial economic burden on the healthcare system through neonatal intensive care costs [3,4]. For women with a previous preterm birth, the recurrence risk ranges from 20% to 40%, which is influenced by gestational age of prior delivery and by clinical factors. This elevated recurrence risk suggests the association with genetic, anatomic, inflammatory, and chronic infectious mechanisms [5,6].

A short cervix (≤25 mm) identified by mid-trimester transvaginal ultrasound is a well-established predictor of spontaneous preterm birth, particularly in women with a prior preterm delivery. This combination constitutes a dual-risk condition, with reported recurrence rates exceeding 50% [7,8]. Cervical shortening reflects premature cervical remodeling, characterized by collagen degradation, alterations in the extracellular matrix, and activation of inflammatory pathways, ultimately leading to weakening of the cervical barrier [9,10].

Preventive strategies for women at high risk have evolved substantially over the past two decades. Vaginal progesterone reduces preterm birth risk before 33 weeks by approximately 45% in women with a cervical length ≤15 mm [11], likely through modulation of uterine quiescence and anti-inflammatory mechanisms [12,13]. Subsequent meta-analyses have confirmed its superiority in women with a short cervix [14]; however, its effectiveness in women with prior preterm birth and absence of cervical shortening remains uncertain, as demonstrated by the OPPTIMUM trial [15]. Another treatment alternative is cervical cerclage, which demonstrates comparable efficacy to vaginal progesterone in women at high risk of preterm birth [16].

Nonetheless, several professional societies, including the ISUOG, SMFM and ACOG, recommend cerclage for women with combined risk factors, specifically those with both prior spontaneous preterm birth and mid-trimester cervical shortening. As a surgical procedure, cerclage is also associated with potential perioperative and procedure-related complications, which should be considered in clinical decision making [17,18].

In this context, the cervical pessary has been proposed as a less-invasive alternative that exerts mechanical effect on the cervix, modifying cervical geometry and redistributing uterine forces to reduce preterm birth risk [19]. The PECEP study (2012) reported a reduction from 27% to 6% before 34 weeks in pregnant women with a short cervix [20]; however, although Alfirevic et al. confirm this reduction in a published study [21], a posterior meta-analysis [22] demonstrated inconsistent findings, possibly attributable to population heterogeneity. Knowledge gaps persist, as few trials specifically address dual-risk populations or evaluate combined mechanical and hormonal interventions while accounting for additional predictors such as maternal comorbidities and prior cervical procedures [23]. A recent trial evaluated the association of cervical pessary and progesterone (200 mg/day) in women with a short cervix, demonstrating modest benefits [24]; however, evidence for dual-risk population remains limited.

Recent evidence suggests that vaginal progesterone alone may not consistently benefit women with a prior spontaneous preterm birth, regardless of cervical length [25], raising uncertainty regarding optimal management for dual-risk populations. Accordingly, the primary objective of this analysis was to evaluate whether the addition of a cervical pessary to vaginal progesterone offers incremental benefits in this dual-risk population [26].

## 2. Methods

### 2.1. Study Design

This study presents a preplanned secondary analysis of data (post hoc analysis) derived from the P5 trial, a multicenter, pragmatic, open-label randomized clinical trial conducted across 17 Brazilian health centers between 2015 and 2019. The original protocol was registered at the Brazilian Clinical Trial Registry (ReBEC) under the number UTN: U1111-1164-2636.

The study was approved by the Research Ethics Committee of the coordinating institution and by all participating local institutional review boards (approval number CAAE 38417114.0.2007.5505, approved date: 27 May 2015). All participants provided written informed consent prior to enrollment, following comprehensive disclosure of study procedures, potential risks, and anticipated benefits.

The adoption of an open-label design was necessitated by the impracticability of adequately masking cervical pessary insertion. To minimize potential bias, standardized protocols for participant follow-up and outcome assessment were implemented, accompanied by systematic training of all research personnel.

Methodological aspects of the primary trial analysis have been detailed in previous publications [24,27]. Briefly, eligible participants were women with singleton pregnancies between 18 + 0 and 22 + 6 weeks of gestation who underwent second-trimester transvaginal ultrasound demonstrating a cervical length ≤30 mm. Exclusion criteria comprised: major fetal anomalies; higher-order multiple gestations; prior cervical cerclage or current use of prophylactic therapies for preterm birth prevention; contraindications to progesterone or pessary use; preterm prelabor rupture of membranes; unexplained vaginal bleeding; active preterm labor; clinical or subclinical genitourinary infections; and documented inability to comply with scheduled outpatient follow-up.

Cervical length screening was performed exclusively through transvaginal ultrasonography utilizing high-frequency transducers (≥5 MHz). All examinations were conducted by certified sonographers adhering to a standardized imaging protocol, with the shortest of three consecutive measurements documented [28,29]. Cervical funneling and intra-amniotic fluid sludge were systematically evaluated and integrated as candidate predictor variables in subsequent multivariable logistic regression models. All participants received 200 mg daily of micronized vaginal progesterone from randomization until 36 weeks of gestation or delivery, whichever occurred first. Therapeutic adherence was systematically monitored at scheduled prenatal visits through participant self-report and medication reconciliation. Women allocated to the intervention group additionally received a silicone cervical pessary inserted under aseptic conditions in an outpatient clinical setting. Appropriate pessary placement was systematically confirmed through immediate post-insertion ultrasonographic evaluation. Participants underwent scheduled evaluations at four-week intervals to assess pessary positioning and identify potential device-related complications. The pessary was routinely removed at 36 weeks of gestation or earlier if clinically indicated.

Cervical pessary removal was performed upon onset of labor, membrane rupture, or when delivery became medically necessary. In addition, premature pessary removal occurred at participant request following withdrawal of informed consent.

Maternal, demographic, obstetric, and clinical data were prospectively collected at enrollment and throughout the study using standardized case report forms. Collected variables comprised: demographic characteristics; comprehensive obstetric and gynecological histories; previous cervical surgical procedures; maternal comorbidities; concurrent medication use; and lifestyle factors. Obstetric surveillance incorporated serial clinical examinations and ultrasonographic assessments, with systematic evaluation of cervical parameters (length and funneling), clinical indicators of preterm labor, and pregnancy-related complications.

The primary outcome was defined as spontaneous preterm birth occurring before 34 completed weeks of gestation. Secondary outcomes comprised preterm birth rates at additional gestational age thresholds, as well as identification of principal variables associated with spontaneous preterm birth (n = 479), irrespective of treatment allocation.

### 2.2. Population Selection and Characterization for the Post Hoc Analysis

#### Analytical Strategy and Study Populations

A two-stage analytical strategy was implemented to ensure methodological rigor, distinctly separating the observational analysis of risk factors from the interventional comparative analysis of therapeutic efficacy.

Step 1: Risk Factor Analysis in a Broad-Risk Cohort

The first stage of the analysis focused on identifying predictors of recurrent spontaneous preterm birth (sPTB). For this purpose, an expanded cohort was utilized, comprising all 479 women with a documented history of prior spontaneous preterm birth who participated in the P5 trial, irrespective of cervical length (CL) or subsequent treatment allocation. This cohort encompassed both women who, presenting with a CL > 30 mm, did not receive any preventive intervention, as well as those who, presenting with CL ≤ 30 mm (n = 155), were randomized to receive active treatment. The objective of this observational analysis was to identify baseline maternal and clinical factors associated with an elevated risk of sPTB recurrence in this comprehensive population of women with documented prior spontaneous preterm delivery.

Step 2: Intervention Efficacy Analysis in High-Risk Cohorts

The second stage was designed to evaluate the comparative efficacy of combined cervical pessary plus vaginal progesterone versus isolated vaginal progesterone. This analysis was restricted to the 155 participants presenting a dual-risk profile: documented history of prior sPTB combined with mid-trimester cervical shortening (CL ≤ 30 mm measured between 18 and 22 weeks of gestation). These women were randomized to one of two treatment arms for a comparative efficacy analysis:

• Intervention Group: Received a combination of a cervical pessary plus vaginal progesterone.
• Control Group: Received vaginal progesterone alone

Within this dual-risk cohort, a further subanalysis was conducted on a very-high-risk subpopulation comprising the 85 women with both documented prior sPTB and more pronounced cervical shortening (CL ≤ 25 mm). This stratification enabled focused evaluation of intervention efficacy in the subgroup at highest risk for early spontaneous preterm delivery. Previous preterm birth was operationally defined as documented delivery between 20 + 0 and 36 + 6 weeks of gestation resulting from spontaneous labor (including premature rupture of the membranes), explicitly excluding elective preterm births due to maternal or fetal indications (medically indicated).

Cervical cerclage procedures were not permitted following randomization; women with pre-existing cerclage were excluded at screening. Consequently, the final analytical cohort contained no participants with cervical cerclage.

Serial participant evaluations comprised systematic assessment of: (1) cervical parameters—longitudinal cervical length, presence and extent of cervical funneling; (2) labor indicators—uterine contractile activity, cervical modifications, and fetal station; (3) pregnancy complications—hypertensive disorders, gestational diabetes mellitus, fetal growth restriction, oligohydramnios, and polyhydramnios.

### 2.3. Primary and Secondary Outcomes

All analyses, encompassing both risk assessment and intervention efficacy evaluation, exclusively focused on spontaneous preterm births. Medically indicated preterm births were systematically excluded from all outcome assessments. The primary outcome of this study was defined as spontaneous preterm birth occurring before 34 weeks of gestation. This specific threshold was selected based on its well-established clinical significance, as delivery before 34 weeks is consistently associated with substantially elevated neonatal morbidity and mortality.

Secondary outcomes encompassed preterm birth rates at additional gestational age thresholds: before 28, 32, and 37 completed weeks of gestation. Additional secondary objectives included identification of significant maternal and clinical predictor variables associated with spontaneous preterm birth recurrence. This predictive analysis was conducted on the expanded cohort of 479 women with documented prior spontaneous preterm birth, irrespective of cervical length measurement or subsequent treatment allocation.

### 2.4. Statistical Analysis

#### 2.4.1. Sample Description

Continuous variables were presented as means ± standard deviations (for normally distributed data) or medians with interquartile ranges (for non-normally distributed data), following verification of normality assumptions using the Kolmogorov–Smirnov test. Categorical variables were expressed through absolute and relative frequencies, with 95% confidence intervals calculated for proportions when clinically relevant.

#### 2.4.2. Group Comparisons

For the comparison of continuous variables between two groups, Student’s *t*-test was applied for normally distributed data, whereas the Mann–Whitney U test was employed for non-normally distributed variables. For comparison of continuous variables across multiple groups, ANOVA or the Kruskal–Wallis test was applied, with appropriate post hoc correction. Categorical variables were compared using Pearson’s chi-square test when expected cell frequencies exceeded 5, or Fisher’s exact test when any expected cell frequency was below 5.

#### 2.4.3. Multivariate Analysis

Independent predictive variables were identified through multivariable logistic regression models employing forward stepwise selection, with entry criterion set at *p* < 0.10 and retention criterion at *p* < 0.05. Candidate variables were selected based on clinical plausibility and results from preliminary bivariate analyses. Biologically plausible two-way interactions between variables were systematically evaluated, and alternative model specifications were compared to optimize goodness-of-fit. Results are expressed as the odds ratio (OR) with corresponding 95% confidence intervals (95% CI). The adequacy of models was evaluated using the Hosmer–Lemeshow goodness-of-fit test, complemented by systematic examination of residuals.

#### 2.4.4. Diagnostic Accuracy Analysis

The discriminatory capacity of predictive models was assessed by receiver operating characteristic (ROC) curve analysis, with calculation of area under the curve (AUC) and corresponding 95% confidence intervals. Optimal cutoff points were determined using Youden’s index, calculating sensitivity, specificity, positive and negative predictive values (PPV and NPV), likelihood ratios, and overall classification accuracy.

An acknowledged limitation of this study was the absence of internal validation for the developed predictive model. Although clinically and biologically plausible criteria guided variable selection, the absence of cross-validation or bootstrapping techniques precluded robust assessment of model generalizability. Consequently, the presented results should be interpreted as exploratory and hypothesis generating, necessitating validation in independent cohorts.

#### 2.4.5. Survival Analysis

Time-to-delivery between treatment groups was compared using Kaplan–Meier survival curves, with statistical testing performed using the log-rank test (for overall survival differences) and Breslow–Wilcoxon test (for early survival differences).

#### 2.4.6. Sample Size and Statistical Power

Sample size for the primary trial was calculated anticipating a reduction in preterm birth rate before 34 weeks from 15% to 8%, with 80% statistical power and a 5% significance level. For the post hoc subgroup analyses, retrospective power calculations were performed, considering the observed effect sizes and available sample sizes within each stratum.

#### 2.4.7. Statistical Software

All analyses were performed using SPSS version 30.0 (IBM Corp., Armonk, NY, USA), with an established significance level of *p* < 0.05 for all comparisons.

#### 2.4.8. Specific Methodological Aspects of Post Hoc Analyses

Although the post hoc analyses were preplanned as secondary objectives of the original trial, they require cautious statistical interpretation. Because the parent study was designed and powered for a broader primary analysis—not for the specific subgroup contrasts reported here—the effective sample sizes and event counts may limit statistical power and precision. Accordingly, these results should be regarded as exploratory and hypothesis-generating. To mitigate the risk of spurious findings, multiplicity adjustments were applied when appropriate (Bonferroni or false discovery rate control). Subgroup stratification followed established, biologically plausible clinical criteria to avoid arbitrary partitions. For analyses involving multiple outcomes, the type I error rate was controlled through conservative procedures to support the robustness of inferences; however, these safeguards cannot fully overcome the inherent power limitations of post hoc subgroup analyses.

## 3. Results

### 3.1. Basic Demographic Characteristics

Demographic characteristics were similar between treatment arms, which confirmed adequate randomization balance. In the subgroup with cervical length ≤30 mm (n = 155; progesterone alone n = 72 vs. pessary plus progesterone n = 83), mean maternal age was 29.3 ± 6.6 vs. 29.1 ± 6.4 years (*p* = 0.922). Mixed ethnicity predominated (43.4% vs. 41.7%), followed by white (38.6% vs. 30.6%), and black (18.1% vs. 26.4%; *p* > 0.05). Mean education attainment was similar between groups: (10.5 ± 2.7 vs. 10.2 ± 2.7 years; *p* = 0.521). Previous obstetric history was comparable, with prior vaginal deliveries reported by 77.1% vs. 83.4% of participants (*p* = 0.499), and previous cesarean sections by 79.5% vs. 77.8% (*p* = 0.594). History of prior miscarriage was present in 44.6% vs. 38.9% of participants (*p* = 0.573). Prevalence of maternal comorbidities was similar (33.7% vs. 26.4%; *p* = 0.348), as was current tobacco use (10.8% vs. 5.6%; *p* = 0.236). Self-reported alcohol consumption differed between groups (7.2% vs. 0%; *p* = 0.02). History of uterine curettage was reported by 31.3% vs. 31.9% (*p* = 0.934); and previous cervical conization by 2.4% vs. 4.2% (*p* = 0.537). Previous delivery of a low-birth-weight infant (<2500 g) occurred in 77.1% vs. 73.6% (*p* = 0.614). In the subgroup with more pronounced cervical shortening (≤25 mm; n = 85), baseline characteristics demonstrated similar balance across treatment arms, with no statistically significant differences in ethnicity distribution, education attainment, or obstetric history variables (*p* > 0.05 for the majority).

### 3.2. Cervical Pessary Efficacy

The efficacy of combined cervical pessary plus progesterone versus progesterone alone was assessed across multiple preterm birth gestational age cutoffs in two distinct high-risk populations. No statistically significant differences in spontaneous preterm birth rates (<37, <34, <32, or <28 weeks of gestation) were observed between the combined and isolated therapy groups. This finding was consistent in women with cervical length ≤30 mm (n = 155; pessary plus progesterone n = 83 vs. progesterone alone n = 72), as detailed in Table 1, and similarly for the subgroup with more pronounced cervical shortening ≤25 mm (n = 85; pessary plus progesterone n = 42, progesterone alone n = 43), with specific data presented in Table 2.

**Figure 1:**
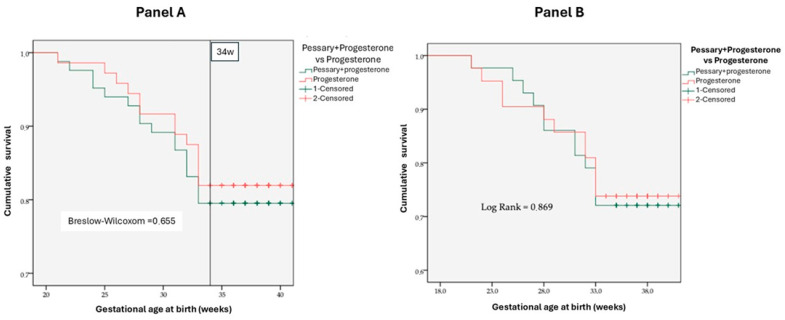
Kaplan–Meier survival curves illustrating the cumulative proportion of women without preterm birth before 3 weeks of gestation. In both panels, the green line represents combined pessary plus progesterone, and the red line represents progesterone alone. The horizontal axis indicates gestational weeks, and the vertical axis shows the proportion without preterm birth. Panel A presents the comparison for the subgroup with cervical length ≤30 mm (Breslow-Wilcoxon test: *p* = 0.655). Panel B displays the comparison for the subgroup with cervical length ≤25 mm (Log-Rank test: *p* = 0.869).

Kaplan–Meier survival curves for preterm birth <34 weeks substantial overlap between treatment groups: *p* = 0.655 (Breslow–Wilcoxon) for ≤30 mm (Panel A); *p* = 0.869 (log-rank test) for ≤25 mm (Panel B) (Figure 1).

### 3.3. Predictive Factors for Recurrent Preterm Birth

**Figure 2:**
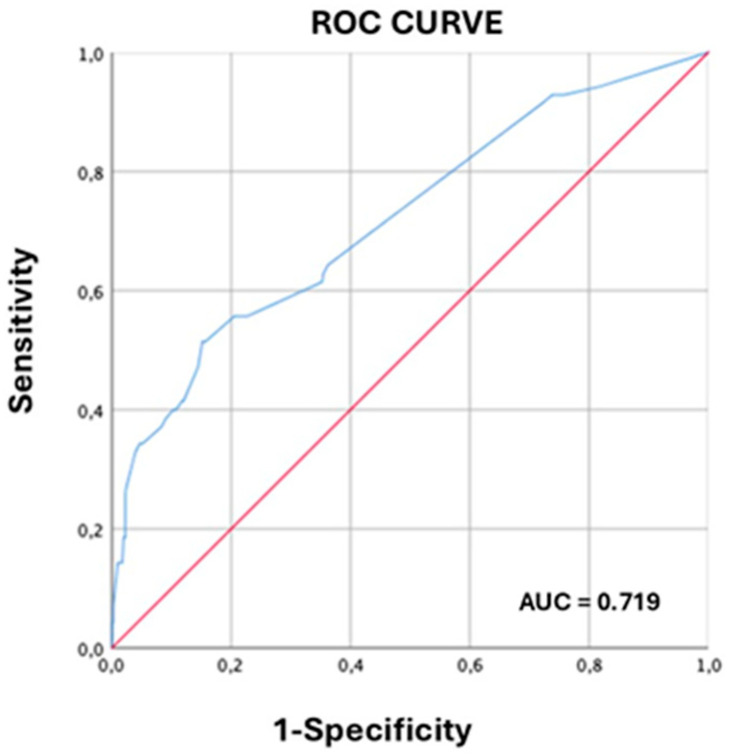
Receiver operating characteristic (ROC) curve for the multivariable predictive model of spontaneous preterm birth before 34 weeks of gestation in women with prior spontaneous preterm delivery (n = 479). Area under the curve (AUC) = 0.719. The horizontal axis represents 1-specificity; the vertical axis represents sensitivity.

Among the 479 women with documented prior spontaneous preterm birth, independent predictors of recurrent preterm delivery before 34 weeks included: higher educational attainment (OR 2.37), previous cervical conization (OR 4.78), previous low birth weight delivery (OR 2.43), previous miscarriages (OR 1.36), twin pregnancy (OR 14.86), and presence of cervical funneling (OR 3.6). The predictive model demonstrated an area under the receiver operating characteristic curve (AUC) of 0.719, with sensitivity of 87.0%, specificity of 58.8%, PPV of 85.7%; and NPV of 98.3% (Table 3, Figure 2).

For the purpose of this study, “higher education” was defined as having completed more than 12 years of schooling or holding a high school diploma. “Previous low birth weight” referred to a history of pregnancies resulting in an infant born with a weight less than 2.5 kg. “Cervical funneling” characterized patients with a cervical length less than 30 mm who also presented with an opening of the internal cervical os greater than 1 cm, described as a finger-like invagination. Lastly, the term “previous conization” encompassed individuals with a history of prior cervical procedures, specifically conization or Loop Electrosurgical Excision Procedure (LEEP).

## 4. Discussion

### 4.1. Synthesis of Main Findings

The results of this study support three fundamental conclusions. First, unlike prior studies conducted in mixed populations, cervical pessary demonstrated no benefit in women with previous preterm birth and a short cervix, suggesting that subgroup presents distinct underlying pathophysiologic mechanisms. Second, the identified variables may contribute to clinically applicable risk stratification tools, enabling a more personalized therapeutic approach. Third, the findings indicate that recurrent preterm birth is driven by multifactorial mechanisms that extend beyond cervical shortening alone, thereby suggesting the need for more comprehensive therapeutic strategies. These results may have important implications for public health policies and clinical protocols, particularly with regard to the presumed universal applicability of cervical interventions in dual-risk populations.

### 4.2. Re-Evaluating Mechanistic Hypotheses for Pessary Non-Efficacy in Recurrent Preterm Birth

The results of this study challenge fundamental assumptions about the cervical pessary’s role in the prevention of preterm birth. The complete absence of benefit in pregnant women with a history of preterm birth, even in those with severe cervical shortening (≤25 mm), suggests a distinct pathophysiologic entity compared to that observed in populations without adverse obstetric history.

This finding has profound implications for understanding the mechanisms underlying recurrent preterm birth. The cervix functions as a barrier between the uterine cavity and vaginal environment, maintaining closure of the internal os until term to enable normal fetal development [10,30]. However, in women with prior preterm birth, cervical competency may be compromised by structural alterations undetectable by conventional ultrasonographic screening methods.

### 4.3. Molecular Mechanisms Underlying Recurrent Cervical Insufficiency

Contemporary research reveals that maternal systemic inflammation and psychosocial stress have been strongly implicated in spontaneous preterm labor, even in the absence of intrauterine infection [31,32]. This finding suggests that in women with prior preterm birth, systemic inflammatory processes may have created an altered biological substrate in which purely mechanical interventions prove insufficient.

The prevailing hypothesis posits that the cervical pessary acts primarily through modification of the cervical–corporal angle and redistribution of mechanical forces on the cervix [33,34,35]. However, if recurrent preterm birth results from early-onset inflammatory cascades, deficiency in maternal immune response, or dysregulation of the fetal hypothalamic-pituitary-adrenal axis, purely anatomical interventions fail to address the fundamental pathophysiologic mechanisms [36,37]. Emerging evidence suggests that women with prior preterm birth may present “inflammatory memory” in cervical and endometrial tissue, maintaining a chronic immunologic activation state that predisposes to recurrent preterm delivery [38]. This concept, still under investigation, could explain why factors such as previous low birth weight (OR 2.43 in our study) and previous miscarriages (OR 1.36) appear as a consistent predictors.

### 4.4. The “Missed Therapeutic Window” Paradigm

An alternative interpretation is that, by the time of pessary insertion (18–22 weeks of gestation), critical pathologic processes have already been irreversibly established in women with prior preterm birth. Unlike women without adverse history, in whom cervical shortening may represent a primarily anatomic and potentially reversible phenomenon, women with prior preterm birth may harbor early biochemical modifications for which intervention at 18–22 weeks represents a missed therapeutic window [3].

Future studies should explore whether earlier pessary insertion, ideally during the first trimester, could demonstrate superior efficacy. Alternatively, multimodal therapeutic strategies combining mechanical support (pessary), hormonal supplementation (progesterone), anti-inflammatory agents, and microbiome modulation (probiotics) may be necessary to address the multifactorial pathophysiologic of recurrent preterm birth. This consideration may partially explain the cerclage success in women with prior preterm birth as cerclage candidates due to cervical shortening, initiate surveillance and undergo intervention earlier, at approximately 16 weeks of gestation [39].

### 4.5. International Comparisons and Population Heterogeneity

Our population differed significantly from the European cohort evaluated by Alfirevic et al. [21]. While their study included 1108 women with a short cervix, only a minority reported prior preterm birth. In contrast, our specific focus on a high-risk subpopulation may explain the lack of therapeutic benefit observed. Another study, also conducted by Alfirevic et al. [23] and conducted across multiple countries in women with short cervix and a history of preterm birth, reported comparable efficacy among progesterone, pessary alone, and cerclage. The findings of the current study demonstrate a similar pattern, with vaginal progesterone appearing to exert the predominant protective effect in populations with dual risk. In this context, the absence of an additional benefit from pessary insertion does not invalidate previously reported positive findings in the broader population [40].

Beyond clinical characteristics, the socioeconomic profiles of our cohort and European population differ substantially. In our population, 69.8% of women had completed high school education, with only 6.9% achieving higher education, contrasting with the typically higher educational attainment reported in European cohorts, which also typically have more universal healthcare access. This difference may reflect not only economic disparities but also distinct patterns of exposure to environmental risk factors, nutritional quality, and prevalence of undiagnosed comorbidities [41,42].

Although high maternal education is conventionally considered a protective factor, the observed association between higher education and increased preterm birth (OR 2.37) in our Brazilian cohort may reflect context-specific socioeconomics mechanisms [43]. First, previous Brazilian research has demonstrated that the private health sector—utilized more frequently by women with higher educational levels—exhibits elevated rates of preterm birth induced by obstetric intervention (adjusted OR ≈ 2.3) [44]. Second, women with higher educational levels may postpone motherhood and be exposed to greater occupational stress or other non-traditional risk factors, potentially neutralizing the protective effect usually attributed to education. Third, this subgroup may demonstrate selective patterns of delayed referral or late presentation to specialized care, resulting in higher rates of obstetric interventions. This phenomenon of “inverse inequality” has already been documented in Brazil [45]. Thus, our finding may represent a specific local epidemiologic pattern, and not a reversal of the classical protective effect.

These predictive models, although promising, reveal inherent limitations due to sample size—smaller subgroups, such as that with n = 85, generate wider confidence intervals, which suggests caution in generalization. Nevertheless, consistency across analyses (e.g., funneling as a recurrent predictor) suggests common pathophysiologic pathways, including prior cervical trauma and chronic inflammation [46]. Compared to international cohorts such as those reported by Alfirevic et al. [21,23], our Brazilian population demonstrated higher prevalence of maternal comorbidities (approximately 30% versus 20–25% in European studies), potentially explaining higher rates of preterm birth (40–46% versus 30–35%), although not altering the lack of pessary efficacy. In conclusion, these results pave the way for a more specific prevention strategies, emphasizing precise identification of women who would benefit from intensive interventions [47].

### 4.6. Implications of Ethnic Diversity in Brazil

The ethnic diversity of our sample, with a predominance of women of mixed race (42.5%) and black ethnicity (22.3%), contrasts with international studies typically conducted in predominantly ethnically homogeneous Caucasian populations. Emerging evidence suggests that genetic polymorphisms may influence both susceptibility to preterm birth and the therapeutic response to specific interventions [48].

### 4.7. Implications for Predictive Modeling and Personalized Care

The predictive models developed in this study (AUC 0.719) demonstrated clinically meaningful discriminatory capacity, comparable to or exceeding many existing risk stratification tools reported in the literature. Beyond simple risk factor identification, this approach enables individualized probabilistic risk quantification, which facilitates personalized therapeutic decisions.

The dual-risk model, demonstrating 87.0% sensitivity and 58.8% specificity, requires external validation in independent cohorts to establish its utility as a screening tool in tertiary referral centers. Women classified as high risk could receive more intensified interventions including cervical cerclage, eventual hospitalization for severe cervical shortening (<5 mm), and home-based activity monitoring, while those at lower risk could be followed with less invasive surveillance protocols.

### 4.8. Limitations and Methodological Perspectives

Despite its robust design as the largest randomized study evaluating patients with dual risk factors (short cervix and prior preterm birth), this study presents several limitations. The open-label design and potential center-to-center variability warrant consideration. The preplanned post hoc analyses inherently had limited statistical power to detect small, clinically relevant effects, given the primary trial’s broader powering. This, compounded by wide confidence intervals—particularly in subgroup analyses—further restricted the precision of findings. Consequently, results should be interpreted as exploratory rather than definitive, often reflecting unstable estimates, and inherent risk of overfitting, attributable to limited sample sizes and lower event rates. Furthermore, the absence of internal validation for the predictive models developed means their generalizability to other populations remains uncertain.

## 5. Conclusions

This study may increase the comprehension of recurrent preterm birth and the limitations of traditional cervical interventions in this high-risk population. The suggestion that pessaries do not benefit women with prior preterm birth challenges established paradigms and opens the path to more sophisticated and personalized approaches, such as cervical cerclage in a dual-risk population with cervix ≤25 mm. The predictive models developed may provide valuable tools for risk stratification that, following prospective external validation, could contribute to more targeted interventions and potentially reduce spontaneous preterm birth rates in high-risk populations.

## Figures and Tables

**Table 1:** Spontaneous preterm birth rates and odds ratios comparing combined cervical pessary plus progesterone versus progesterone alone in women with cervical length ≤30 mm (n = 155).

Outcome	Progesterone Alone [% (n)] (n = 72)	Pessary + Progesterone [% (n)] (n = 83)	OR (CI 95%)	*p*-Value
<28 weeks	5.6 (4)	7.2 (6)	1.325 (0.359–4.892)	0.672
<32 weeks	11.1 (8)	13.3 (11)	1.222 (0.463–3.227)	0.685
<34 weeks	18.1 (13)	20.5 (17)	1.169 (0.524–2.609)	0.703
<37 weeks	40.3 (29)	39.8 (33)	0.979 (0.514–1.864)	0.948

**Table 2:** Spontaneous preterm birth rates and odds ratios comparing combined cervical pessary plus progesterone versus progesterone alone in women with cervical length ≤25 mm (n = 85).

Outcome	Progesterone Alone [% (n)] (n = 43)	Pessary + Progesterone [% (n)] (n = 42)	OR (CI 95%)	*p*-Value
<28 weeks	9.3 (4)	9.5 (4)	1.500 (0.392–5.733)	0.551
<32 weeks	18.6 (8)	14.3 (6)	0.946 (0.320–2.795)	0.920
<34 weeks	27.9 (12)	26.2 (11)	1.167 (0.466–2.921)	0.742
<37 weeks	46.5 (20)	45.2 (19)	1.100 (0.476–2.541)	0.823

**Table 3:** Multivariable logistic regression analysis: independent predictors of spontaneous preterm birth before 34 weeks of gestation in women with prior spontaneous preterm delivery (n = 479). Model performance: area under the receiver operating characteristic curve (AUC) = 0.719.

Predictor	OR	CI 95%	*p*-Value
Higher education	2.37	0.99–5.63	0.024
Previous conization	4.78	1.08–21.19	0.039
Previous low birth weight	2.43	1.22–4.85	0.051
Previous miscarriages	1.36	1.10–1.69	0.005
Twin pregnancy	14.86	4.35–50.68	<0.001
Cervical funneling	3.6	1.79–7.24	<0.001

## Data Availability

De-identified individual participant data (IPD) are available from the corresponding author upon reasonable request and execution of a Data Sharing Agreement for non-commercial academic use, subject to ethics approval and institutional data-transfer policies.
